# The Protein Secretome Is Altered in Rectal Cancer Tissue Compared to Normal Rectal Tissue, and Alterations in the Secretome Induce Enhanced Innate Immune Responses

**DOI:** 10.3390/cancers13030571

**Published:** 2021-02-02

**Authors:** Aisling B. Heeran, Margaret R. Dunne, Maria E. Morrissey, Croí E. Buckley, Niamh Clarke, Aoife Cannon, Noel E. Donlon, Timothy S. Nugent, Michael Durand, Cara Dunne, John O. Larkin, Brian Mehigan, Paul McCormick, Niamh Lynam-Lennon, Jacintha O’Sullivan

**Affiliations:** 1 Trinity St. James’s Cancer Institute, Trinity Translational Medicine Institute, Department of Surgery, Trinity College Dublin and St. James’s Hospital, D08 W9RT Dublin 8, Ireland; heerana@tcd.ie (A.B.H.); dunnem12@tcd.ie (M.R.D.); miamorrissey@gmail.com (M.E.M.); bucklecr@tcd.ie (C.E.B.); clarken1@tcd.ie (N.C.); cannona@tcd.ie (A.C.); donlonn@tcd.ie (N.E.D.); nugentti@tcd.ie (T.S.N.); lynamlen@tcd.ie (N.L.-L.); 2 GEMS, St. James’s Hospital, D08 NHY1 Dublin 8, Ireland; durandm@tcd.ie (M.D.); cardunne@stjames.ie (C.D.); jlarkin@stjames.ie (J.O.L.); bmehigan@stjames.ie (B.M.); pmccormick@stjames.ie (P.M.)

**Keywords:** rectal cancer, dendritic cells, inflammation, tumour immunology, radiotherapy

## Abstract

**Simple Summary:**

Rectal cancer occurs in the lower part of the bowel, and approximately half of all rectal cancer patients receive chemoradiotherapy before surgery. In ~22% of cases the tumour is eradicated, but the reasons for different response rates between patients are largely unknown. Inflammation and the immune system are important players in the response to cancer treatment, but we do not fully understand the role they play in this clinical setting. We examined the levels of 54 inflammatory markers in normal (non-cancerous) rectal tissue and rectal cancer tissue, and we found that rectal cancer tissue was more inflammatory, and the levels of inflammatory markers correlated with obesity status. We found that irradiating rectal cancer tissue enhanced the ability of immune cells to induce an anti-tumour immune response.

**Abstract:**

Locally advanced rectal cancer is treated with neoadjuvant-chemoradiotherapy; however, only ~22% of patients achieve a complete response, and resistance mechanisms are poorly understood. The role of inflammation and immune cell biology in this setting is under-investigated. In this study, we profiled the inflammatory protein secretome of normal (non-cancer) (*n* = 8) and malignant rectal tissue (*n* = 12) pre- and post-radiation in human ex vivo explant models and examined the influence of these untreated and treated secretomes on dendritic cell biology (*n* = 8 for cancer and normal). These resultant profiles were correlated with patient clinical characteristics. Nineteen factors were secreted at significantly higher levels from the rectal cancer secretome when compared to the normal rectal secretome; Flt-1, P1GF, IFN-γ, IL-6, IL-10, CCL20, CCL26, CCL22, CCL3, CCL4, CCL17, GM-CSF, IL-12/IL-23p40, IL-17A, IL-1α, IL-17A/F, IL-1RA, TSLP and CXCL10 (*p* < 0.05). Radiation was found to have differential effects on normal rectal tissue and rectal cancer tissue with increased IL-15 and CCL22 secretion following radiation from normal rectal tissue explants (*p* < 0.05), while no significant alterations were observed in the irradiated rectal cancer tissue. Interestingly, however, the irradiated rectal cancer secretome induced the most potent effect on dendritic cell maturation via upregulation of CD80 and PD-L1. Patient’s visceral fat area correlated with secreted factors including CCL20, suggesting that obesity status may alter the tumour microenvironment (TME). These results suggest that radiation does not have a negative effect on the ability of the rectal cancer TME to induce an immune response. Understanding these responses may unveil potential therapeutic targets to enhance radiation response and mitigate normal tissue injury. Tumour irradiation in this cohort enhances innate immune responses, which may be harnessed to improve patient treatment outcome.

## 1. Introduction

Rectal cancer is a malignancy that occurs in the lower part of the large intestine. Approximately 700,000 cases of rectal cancer are diagnosed globally each year, and the annual death toll is approximately 310,000 [1]. The standard of care for locally advanced rectal cancer is neoadjuvant chemoradiotherapy (neo-CRT) [2]. While ~40–60% of patients achieve some level of downstaging following neoadjuvant treatment [3], only 15–27% of these patients achieve a complete pathological response to treatment [4], meaning the potential for cure in the remaining patients depends on resectional surgery with its attendant morbidity and long-term functional implications. The role of the tumour microenvironment (TME) and the biological mechanisms underlying these responses to treatment are poorly understood.

The TME describes the milieu of cancer cells, infiltrating immune cells, secreted factors and the extracellular matrix. The interaction between the tumour cells and the surrounding microenvironment profoundly affects tumour progression and treatment response [5]. Much work to date has investigated the colorectal cancer (CRC) TME, and while the colon and rectum are anatomically related, recurrence rates [6] and treatment regimens differ between the cancer types [7]. The interplay between the TME, secreted inflammatory mediators and immune cell function and how it may be altered by radiation is poorly understood in the context of rectal cancer. Furthermore, to fully understand the alterations occurring in the rectal cancer microenvironment and its interaction with the immune system, it is important to also gain an understanding of the microenvironment in a non-cancerous rectal tissue model.

Tumour-promoting inflammation is a recognised hallmark of cancer [8]. It is postulated that chronic inflammation plays a role in the development of CRC, and anti-inflammatory therapies can reduce risk [9,10]. Secreted factors from the TME may both positively and negatively impact on cancer development. Radiation is known to induce alterations in secreted factors from skin tissue [11]; however, the differential effects of clinically relevant doses of radiation on the normal rectal tissue secretome compared to the rectal cancer tissue secretome are unknown. To date, a comprehensive profiling of the rectal cancer protein secretome and the normal rectal secretome has not been conducted.

The importance of the immune system and its role in carcinogenesis is pivotal [12], and evidence supports the role of the immune system in the radiation response [13]. Dendritic cells (DCs) are professional antigen-presenting cells that reside in blood and tissues in an immature state. Their main function is to recognise pathogens, capture, process and present antigens to T cells to elicit an antigen-specific immune response [14]. DCs are essential for an anti-tumour immune response, and it has been reported that DCs in patients with cancer are incapable of launching a sufficient anti-tumour response [14]. It is becoming increasingly evident that radiation may alter the immune system and inflammatory pathways [15]. Historically, radiation was considered immunosuppressive, with the radiosensitivity of lymphocytes being the dominant explanation for this; however, in recent years radiation is considered immunomodulatory [16]. It is thought that radiation induces innate receptor signalling and subsequent maturation of DCs through tumour cell death and release of endogenous toll-like receptor (TLR) agonists [16]. Furthermore, injection of immature DCs into an irradiated tumour site induced strong tumour-specific cytotoxic T lymphocyte activity in a poorly immunogenic mouse tumour model [17].

In this study we have profiled for the first time the protein secretome of both normal (non-cancer) rectal tissue and rectal cancer tissue pre- and post-radiation, and we examined how these secretomes affect the innate immune system, specifically DC maturation. We hypothesise that the protein secretome will differ between normal (non-cancer) and cancer tissue and that radiation will affect these secretomes and will likely induce them to interact differently with the innate immune system.

## 2. Results

### 2.1. The Protein Secretome Differs between Normal Rectal Tissue and Rectal Cancer Tissue

**Figure 1:**
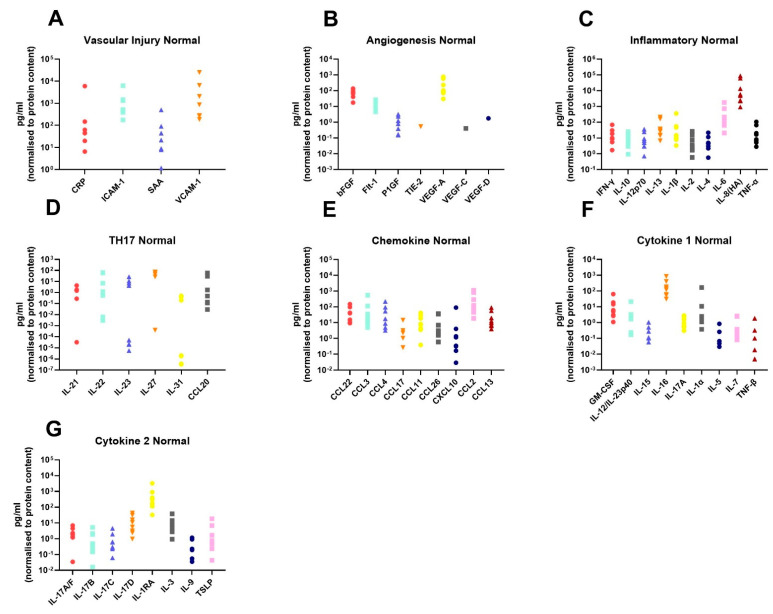
Secreted levels of 53 proteins from *n* = 8 normal rectal biopsies. Normal rectal biopsies were cultured for 24 h, and the resultant NCM was screened for the expression of 54 inflammatory mediators using a multiplex ELISA. (**A**) Secreted levels of vascular injury proteins CRP, ICAM-1, SAA and VCAM-1. (**B**) Secreted levels of angiogenic factors bFGF, Flt-1, P1GF, Tie-2, VEGF-A, VEGF-C and VEGF-D. (**C**) Inflammatory protein secretions IFN-γ, IL-10, IL-12p70, IL-13, IL-1β, IL-2, IL-4, IL-6, IL-8(HA) and TNF-α. (**D**) Secretion of Th17 proteins IL-21, IL-22, IL-23, IL-27, IL-31 and CCL20. (**E**) Chemokine secreted proteins CCL22, CCL3, CCL4, CCL17, CCL11, CCL26, CXCL10, CCL2 and CCL13. (**F**) Secretion of cytokine panel 1 proteins GM-CSF, IL-12/IL-23p40, IL-15, IL-16, IL-17A, IL-1α, IL-5, IL-7 and TNF-β. (**G**) Secretion of cytokine 2 panel proteins IL-17A/F, IL-17B, IL-17C, IL-17D, IL-1RA, IL-3, IL-9 and TSLP. All protein secretions are normalised to protein content of the biopsies.

**Figure 2:**
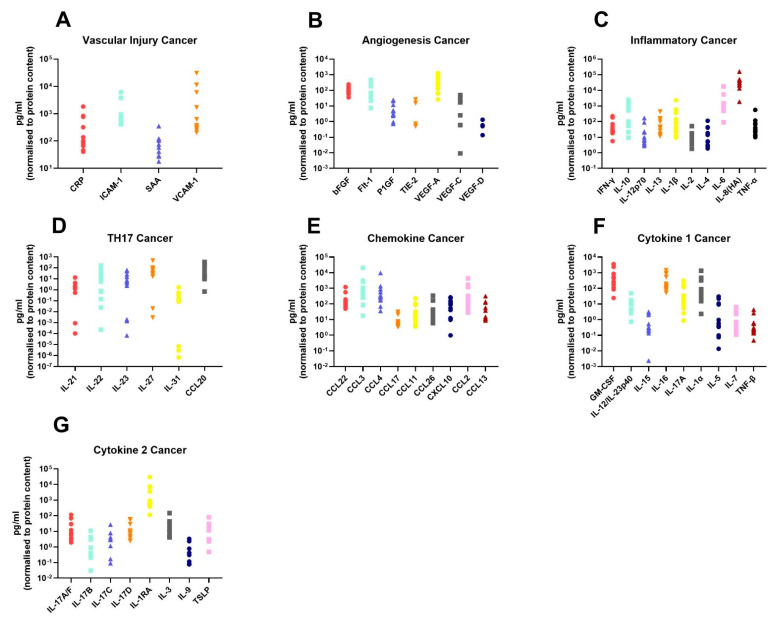
Secreted levels of 53 proteins from *n* = 12 rectal cancer biopsies. Rectal cancer biopsies were cultured for 24 h, and the resultant TCM was screened for the expression of 54 inflammatory mediators using a multiplex ELISA. (**A**) Secreted levels of vascular injury proteins CRP, ICAM-1, SAA and VCAM-1. (**B**) Secreted levels of angiogenic factors bFGF, Flt-1, P1GF, Tie-2, VEGF-A, VEGF-C and VEGF-D. (**C**) Inflammatory secreted proteins IFN-γ, IL-10, IL-12p70, IL-13, IL-1β, IL-2, IL-4, IL-6, IL-8(HA) and TNF-α. (**D**) Secretion of Th17 proteins IL-21, IL-22, IL-23, IL-27, IL-31 and CCL20. (**E**) Chemokine secreted proteins CCL22, CCL3, CCL4, CCL17, CCL11, CCL26, CXCL10, CCL2 and CCL13. (**F**) Secretion of cytokine panel 1 proteins GM-CSF, IL-12/IL-23p40, IL-15, IL-16, IL-17A, IL-1α, IL-5, IL-7 and TNF-β. (**G**) Secretion of cytokine 2 panel proteins IL-17A/F, IL-17B, IL-17C, IL-17D, IL-1RA, IL-3, IL-9 and TSLP. All protein secretions are normalised to protein content of the biopsies.

In order to assess the differences in the inflammatory secretome between normal rectal tissue and rectal cancer tissue, we cultured 12 rectal cancer biopsies and 8 normal rectal biopsies for 24 h and used a multiplex ELISA platform to quantify the expression of 54 inflammatory proteins in the resultant normal conditioned media (NCM) and tumour conditioned media (TCM). The secreted proteins were categorised into 7 panels: vascular injury, angiogenesis, inflammatory, Th17, chemokine, cytokine 1 and cytokine 2. Levels of the secreted factors in normal rectal tissue are displayed in Figure 1A–G, and levels of the secreted factors in rectal cancer tissue are shown in Figure 2A–G. There was notable heterogeneity in levels of secreted factors between patients in both cohorts. Fifty-three of the secreted factors were within the limit of detection for at least one sample in normal rectal biopsies and rectal cancer biopsies. However, three factors—Tie-2, VEGF-C and VEGF-D—were only detectable in one sample each in the normal (non-cancer) cohort. 

**Figure 3:**
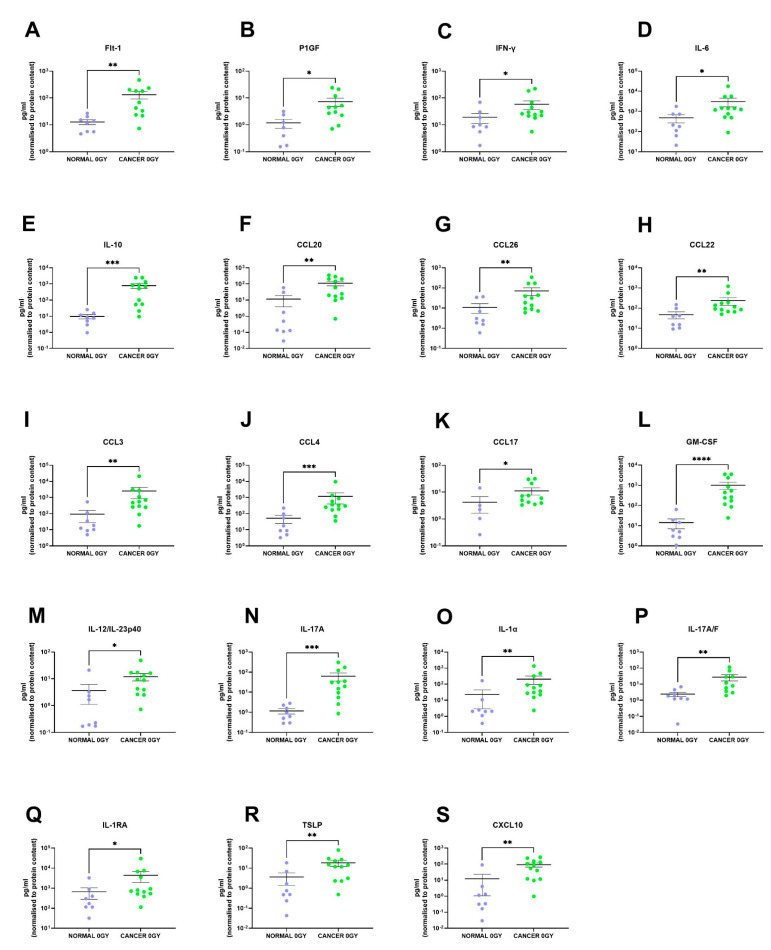
The protein secretome differs between rectal cancer tissue and normal rectal tissue. NCM and TCM from cultured normal rectal and rectal cancer biopsies were screened for the expression of 54 inflammatory secretions using a multiplex ELISA. Nineteen factors were secreted at significantly higher levels in the TCM compared to the NCM. There were significantly higher levels of (**A**) Flt-1, (**B**) P1GF, (**C**) IFN-γ, (**D**) IL-6, (**E**) IL-10, (**F**) CCL20, (**G**) CCL26, (**H**) CCL22, (**I**) CCL3, (**J**) CCL4, (**K**) CCL17, (**L**) GM-CSF, (**M**) IL-12/IL-23p40, (**N**) IL-17A, (**O**) IL-1α, (**P**) IL-17A/F, (**Q**) IL-1RA, (**R**) TSLP and (**S**) CXCL10 in the rectal cancer secretome. All data expressed as mean ± SEM. Statistical analysis by Mann Whitney U-test. *n* = 8 for normal, *n* = 12 for cancer, *n* = 7 normal for P1GF, *n* = 5 normal for CCL17, *n* = 11 cancer for Flt-1, P1GF, CCL17 and *n* = 10 cancer for IL-17A/F. **** *p* < 0.0001, *** *p* < 0.001, ** *p* < 0.01, * *p* < 0.05.

We identified 19 factors that were secreted at significantly higher levels in the rectal cancer secretome compared to the normal rectal secretome: Flt-1 (*p* = 0.001), P1GF (*p* = 0.01), IFN-γ (*p* = 0.04), IL-6 (*p* = 0.02), IL-10 (*p* = 0.0002), CCL20 (*p* = 0.005), CCL26 (*p* = 0.009), CCL22 (*p* = 0.007), CCL3 (*p* = 0.002), CCL4 (*p* = 0.0007), CCL17 (*p* = 0.02), GM-CSF (*p* < 0.0001), IL-12/IL-23p40 (*p* = 0.01), IL-17A (*p* = 0.0003), IL-1α (*p* = 0.003), IL-17A/F (*p* = 0.003), IL-1RA (*p* = 0.03), TSLP (*p* = 0.007) and CXCL10 (*p* = 0.001) (Figure 3A–S). This indicates that the rectal cancer secretome is more inflammatory than the normal rectal secretome, which is expected since tumour-promoting inflammation is a hallmark of cancer [8].

### 2.2. Radiation Significantly Altered the Secretion of IL-15 and CCL22 from Normal Rectal Tissue

**Figure 4:**
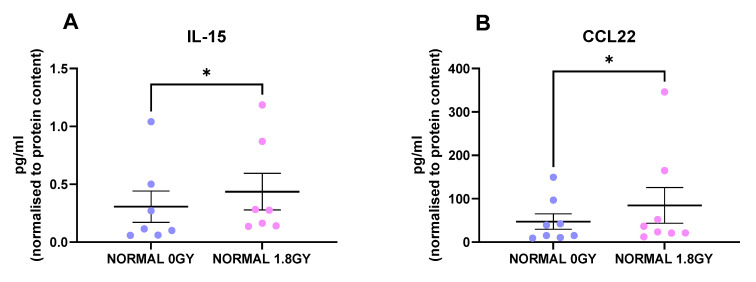
Radiation alters the secretome of normal rectal tissue. NCM from mock-irradiated and irradiated normal rectal biopsies were screened for the secretion of 54 inflammatory proteins using a multiplex ELISA. Following a single fraction of 1.8 Gy radiation, there were significantly higher levels of (**A**) IL-15 and (**B**) CCL22 in the normal rectal secretome. All data expressed as mean ± SEM. Statistical analysis by Wilcoxon signed-rank test. *n* = 8 for CCL22 and *n* = 7 for IL-15, * *p* < 0.05.

Radiation is the standard of care for locally advanced rectal cancer, with about 50% of rectal cancer patients receiving this treatment modality; therefore, we investigated the effect of a clinically relevant dose of 1.8 Gy radiation on protein secretions from normal rectal tissue and rectal cancer tissue. Interestingly, we found 2 factors that were secreted at significantly higher levels from irradiated normal rectal tissue compared to mock-irradiated normal rectal tissue: IL-15 (*p* = 0.01) and CCL22 (*p* = 0.03) (Figure 4A,B). This indicates that a single fraction of 1.8 Gy radiation alters levels of secreted proteins in normal rectal tissue. We did not observe any significant alterations in secreted factors in rectal cancer tissue following a single fraction of a clinically relevant dose of radiation. This suggests that a single fraction of 1.8 Gy radiation does not alter secreted proteins in rectal cancer tissue.

### 2.3. The Rectal Cancer Microenvironment Alters Expression of Maturation Markers on CD11c^+^ Dendritic Cells

**Figure 5:**
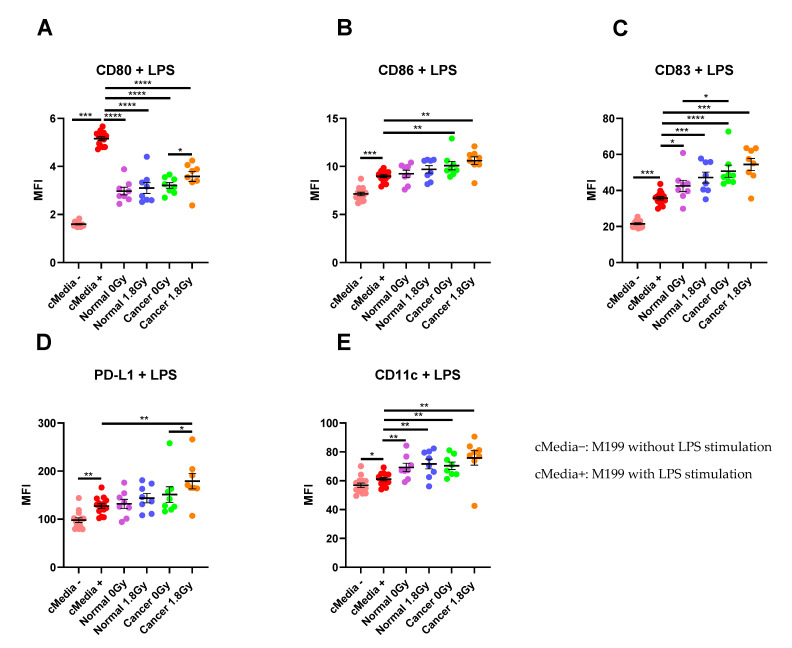
The effect of NCM and TCM on LPS-induced dendritic cell (DC) maturation. CD11c^+^ DCs were treated with NCM and TCM from mock-irradiated and irradiated biopsies, and the effect on DC maturation markers was assessed by flow cytometry. (**A**) NCM and TCM from both mock-irradiated and irradiated biopsies significantly inhibit LPS-induced expression of CD80. TCM from irradiated rectal cancer tissue has a less inhibitory effect than TCM from mock-irradiated rectal cancer tissue. (**B**) TCM from both irradiated and mock-irradiated rectal cancer tissue caused significant enhancement of LPS-induced expression of CD86. (**C**) NCM and TCM from both irradiated and mock-irradiated normal rectal and rectal cancer biopsies caused a significant enhancement of LPS-induced expression of CD83. TCM from mock-irradiated rectal cancer biopsies caused significant elevation of CD83 expression levels compared to NCM from mock-irradiated biopsies. (**D**) PD-L1 expression was significantly enhanced by TCM from irradiated rectal cancer tissue. (**E**) LPS-induced expression of CD11c was significantly elevated by NCM and TCM from irradiated and mock-irradiated biopsies. All data expressed as mean ± SEM. Statistical analysis by Wilcoxon signed-rank test when comparing the same tissue type, e.g., Cancer 0 Gy vs. Cancer 1.8 Gy and Mann Whitney U-test when comparing different tissue types and comparing to media control. *n* = 14 for cMedia, *n* = 8 for normal and cancer, **** *p* < 0.0001, *** *p* < 0.001, ** *p* < 0.01, * *p* < 0.05.

To assess the interaction between the TME and the immune system, we examined the effect of TCM and NCM from both mock-irradiated and irradiated biopsies on DC maturation markers. Following lipopolysaccharide (LPS)-induced maturation of DCs, there was a significant increase in expression of the maturation markers CD80, CD86, CD83 and PD-L1 and the phenotypic marker CD11c (*p* < 0.05). Therefore, we assessed the effects of TCM and NCM from both mock-irradiated and irradiated tissue on these markers. TCM and NCM from both mock-irradiated and irradiated biopsies had a significant inhibitory effect on LPS-induced expression of CD80 (*p* < 0.0001 for all comparisons). However, TCM from irradiated rectal cancer biopsies had the least inhibitory effect on CD80 levels, inducing significantly higher expression of CD80 compared to TCM from mock-irradiated rectal cancer (*p* = 0.03). TCM from both mock-irradiated (*p* = 0.007) and irradiated (*p* = 0.001) rectal cancer tissue significantly enhanced LPS-induced expression of CD86. NCM did not affect expression of CD86 compared to the media only control with LPS (*p >* 0.05). CD83 expression was significantly enhanced by NCM from both mock-irradiated (*p* = 0.01) and irradiated (*p* = 0.0006) biopsies and TCM both mock-irradiated (*p* < 0.0001) and irradiated (*p* = 0.0003) biopsies compared to LPS-induced stimulation. Moreover, TCM from mock-irradiated rectal cancer tissue enhanced expression of CD83 to a greater extent than NCM from mock-irradiated normal rectal tissue (*p* = 0.02). PD-L1 expression levels were significantly elevated on DCs treated with TCM from irradiated rectal cancer tissue (*p* = 0.002) compared to LPS-induced expression. PD-L1 expression was significantly higher on DCs treated with TCM from irradiated tissue compared to TCM from mock-irradiated tissue (*p* = 0.02). Levels of the phenotypic marker CD11c were significantly elevated on DCs exposed to NCM from mock-irradiated (*p* = 0.007) and irradiated (*p* = 0.009) and TCM from both mock-irradiated (*p* = 0.002) and irradiated (*p* = 0.002) rectal cancer tissue (Figure 5A–E). This suggests that the irradiated rectal cancer microenvironment exerts the most potent effect on upregulating DC maturation markers compared to the other three microenvironments investigated. Similar results were observed in the unstimulated setting without LPS whereby the irradiated rectal cancer TME had the most potent stimulatory effect on DC maturation markers (Figure S1).

### 2.4. Linking Innate Immune Response with Secreted Factors in the Normal and Cancer Microenvironments

Given the observed differences in DC maturation markers between CD11c^+^ DCs treated with the normal and cancerous rectal tissue microenvironments, we correlated DC maturation markers in both microenvironments pre- and post-radiation with secreted factors from their respective microenvironment. There were no significant correlations between secreted factors in the mock-irradiated normal rectal microenvironment and markers of DC maturation. There was a significant inverse correlation between IL-10 and CD80 (r = −0.8571, *p* = 0.01) in the irradiated normal microenvironment (Table 1). In the mock-irradiated cancer microenvironment, there was a significant correlation between CD80 and Flt-1 (r = 0.7857, *p* = 0.04), and an inverse correlation between CD80 and IL-27 (r = −0.8214, *p* = 0.03). CD83 correlated with levels of SAA (r = 0.7857, *p* = 0.04). Expression of CD80 on DCs treated with TCM from irradiated rectal cancer tissue correlated with levels of ICAM-1 (r = 0.8214, *p* = 0.03) and CD11c correlated with IL-1RA (r = 0.7619, *p* = 0.03) (Table 2). These results indicate a differential relationship between DC maturation markers and secreted factors in the normal and malignant rectal microenvironments and that radiation further alters this relationship. 

### 2.5. Linking DC Maturation and Phenotypic Markers with Secretions from DCs Treated with TCM and NCM

We performed multiplex ELISA screening on the DC-TCM/NCM supernatants of the DCs treated with TCM and NCM from mock-irradiated and irradiated rectal tissue. We then correlated these factors with DC maturation and phenotypic markers to identify if there was a differential response of DCs to TCM and NCM and if radiation altered this response. ICAM-1 was inversely correlated with CD83 (r = −0.7857, *p* = 0.02) and CD11c (r = −0.7857, *p* = 0.02) on DCs treated with NCM from irradiated normal rectal tissue (Table 3). We found a significant correlation between PD-L1 on DCs treated with TCM from mock-irradiated rectal cancer tissue and TNF-α levels in the supernatants of DCs treated with TCM from mock-irradiated rectal cancer tissue (r = 0.8333, *p* = 0.01). There was an inverse correlation between VEGF-C and CD86 (r = −0.9276, *p* = 0.01) and CD11c (r = −0.8857, *p* = 0.03) in DCs treated with TCM from irradiated rectal cancer tissue (Table 4). This highlights the differential response of DCs to factors in their microenvironment.

### 2.6. Linking Clinical Characteristics with Secreted Factors and DC Maturation and Phenotypic Markers

Given the heterogeneity in the levels of secreted factors in the TME, and to further explore the relationship between factors secreted from the rectal cancer microenvironment, we correlated patient clinical characteristics with secreted factors both pre- and post-radiation. In the mock-irradiated rectal cancer microenvironment, there were several factors that significantly correlated with body composition parameters. Skeletal muscle correlated inversely with Flt-1 (r = −0.6273, *p* = 0.04) and correlated positively with IL-12/IL-23p40 (r = 0.6573, *p* = 0.02), IL-1α (r = 0.5874, *p* = 0.04) and VEGF-A (r = 0.6224, *p* = 0.03). Visceral fat area (VFA) correlated with CCL20 (r = 0.6783, *p* = 0.01) (Table 5) (Figure S2). In the irradiated rectal cancer secretome there was again a significant inverse correlation between skeletal muscle and Flt-1 (r = −0.7182, *p* = 0.01) and VEGF-D (r = −1, *p* = 0.01). Intermuscular fat correlated with CCL20 (r = 0.7133, *p* = 0.01), VEGF-A (r = 0.6084, *p* = 0.03) and IL-1RA (r = 0.6084, *p* = 0.03), and VFA correlated with CCL20 (r = 0.6643, *p* = 0.02) and IL-1RA (r = 0.6503, *p* = 0.02) (Table 6) (Figure S3).

Expression of CD11c on DCs treated with TCM from irradiated rectal cancer tissue correlated significantly with VFA (r = 0.8095, *p* = 0.02) and intermuscular fat (r = 0.7381, *p* = 0.04). These data suggest that obesity may influence the microenvironment of rectal cancer tissue and the response of this microenvironment to radiation therapy. We analysed secreted factor data and DC maturation data according to T and N stage. VEGF-A was significantly reduced in the TCM from irradiated rectal cancer tissue from patients with node positive disease. TSLP was significantly higher in the irradiated TME of patients with T1/2 stage tumours compared to those with T3/4 stage tumours. CD11c was significantly elevated on DCs treated with TCM from irradiated rectal cancer biopsies from patients with node negative disease (data not shown).

## 3. Discussion

The objective of this study was to profile the inflammatory secretome of human rectal cancer and normal rectal tissue pre- and post-radiation and to investigate the interaction between this secretome and the innate immune system, specifically DC maturation markers. By conducting this study, we identified associations between factors within the TME and DC maturation markers, thus providing an avenue for further exploration to harness the therapeutic potential of modulating the TME. Using human ex vivo explants as a model system, we have shown that the protein secretome differs between normal rectal and rectal cancer tissue, and this is altered following radiation in normal rectal tissue, highlighting variation in effects of radiation on the normal compared to the malignant rectal tissue microenvironments. Furthermore, these protein secretome microenvironments interacted differentially with the innate immune system.

We identified 19 factors that were secreted at significantly higher levels in the TME of rectal cancer tissue compared to normal rectal tissue. This indicates that the rectal cancer microenvironment is more inflammatory than the normal rectal microenvironment. Following radiation, two factors were elevated in the secretome of normal rectal tissue, while surprisingly no secreted factor was significantly altered in malignant rectal tissue following radiation. This indicates that malignant and normal rectal tissue respond differently to radiation and suggests that a single fraction of 1.8 Gy radiation induces alterations in inflammatory secretions in normal tissue but not in malignant tissue. Following exposure of DCs to NCM and TCM from both mock-irradiated and irradiated biopsies, we found that the irradiated rectal cancer secretome caused an enhancement in expression of DC maturation markers. These results suggest that a single fraction of a clinically relevant dose of radiation does not have a negative impact on the inflammatory milieu of malignant rectal tissue and does not lead to suppression of the innate immune system.

The inflammatory environment differs between non-cancer and cancer tissue, which is unsurprising given that tumour-promoting inflammation is a hallmark of cancer [8]. Nineteen out of 54 factors quantified were found at significantly higher levels in the cancer secretome compared to the normal secretome. These factors have previously been reported to have pro-oncogenic attributes including angiogenic, pro-mitogenic and immunosuppressive characteristics; therefore, it is expected that they are elevated in the rectal cancer TME compared to the normal microenvironment, while other elevated factors have anti-tumour functions. Two factors, Flt-1 and P1GF, are angiogenic mediators. P1GF is capable of binding to Flt-1 and may displace VEGF, thereby facilitating enhanced VEGF-activity [18]. P1GF is known to have numerous roles in carcinogenesis including macrophage activation and recruitment, lymph vessel growth, DC suppression, tumour cell proliferation and migration and endothelial cell vessel growth [19]. IL-1α secretion from CRC cells has also been shown to have angiogenic properties [20]. The inflammatory cytokines IFN-γ, IL-6 and IL-10 were also found at significantly higher levels in the cancer secretome compared to the normal secretome. IFN-γ is an important effector of anti-tumour immunity, but more recent evidence suggests that it may play important roles in tumour progression and immune evasion [21]. IL-6 is a well-known pro-tumorigenic factor [22], and IL-10 was originally identified as a potent anti-inflammatory cytokine; however, it is now known that spatial and temporal regulation of IL-10 bestows on it anti-tumour responses also [23].

Several Th17-type proteins including CCL20, IL-17A and IL-17A/F are secreted at higher levels from rectal cancer tissue compared to normal rectal tissue. This is unsurprising given the known involvement of Th17 cells in CRC. Th17 cells infiltrate CRCs, and their density correlates with poor prognosis [24,25]. In multiple models of CRC, antagonising Th17 cytokines exert anti-cancer effects, but there have been mixed reports about the effects of blocking Th17 cytokines in murine models [26]. CCL20 is a chemokine involved in the recruitment of Th17 cells to the TME [27,28].

A number of the elevated proteins in the rectal cancer environment are implicated in tumour immune evasion through recruitment of regulatory T cells (T regs) to the TME. CCL22, CCL17, TSLP and CXCL10 function in recruitment of T regs, with levels of CCL22 and CCL17 correlating with T reg infiltration in gastrointestinal cancers [29,30]. TSLP production by cancer-associated fibroblasts in pancreatic cancer induces Th2 type inflammation and is associated with poorer patient outcome [31]. CXCL10 has been associated with an immunosuppressive phenotype in pancreatic cancer [32].

Two factors, CCL22 and IL-15, were secreted at significantly higher levels following radiation in normal rectal tissue. CCL22 is involved in recruitment of T regs through its receptor CCR4 and is therefore reported as being immunosuppressive [33]. IL-15 is an immunostimulatory cytokine and is involved in development, differentiation and survival of natural killer cells [34]. Therefore, our data suggest that a single fraction of 1.8 Gy radiation may alter the microenvironment of normal rectal tissue to promote an inflammatory response but that homeostatic mechanisms may be at play in limiting the extent of the inflammation. Surprisingly, no factor was significantly altered in rectal cancer tissue following radiation. Undoubtedly, the cellular composition of normal rectal tissue and rectal cancer tissue differs, as does the inflammatory secretome; therefore, it is reasonable to hypothesise that the response to radiation would also differ. When taken in combination with our results on DC maturation markers, whereby the irradiated rectal cancer TME caused enhancement in expression of DC maturation markers relative to the normal microenvironment, these data suggest that irradiating the TME with a single fraction of 1.8 Gy does not have an inflammation-induced immunosuppressive effect. It has been reported that chemotherapy-induced inflammation may contribute to treatment resistance in cancer [35], and enhanced inflammation has been implicated in poor treatment response in patients [36,37]. Taken together, we hypothesise that this is a positive result since an enhancement of inflammation and tumour suppressive mechanisms would have negative consequences on the anti-tumour immune response. However, a limitation of this study was the use of a single fraction of 1.8 Gy radiation. Comparison of the inflammatory secretome of rectal cancer tissue obtained at surgical resection from treatment-naïve patients and those that received a full course of neo-CRT would offer greater insight into the effect of clinical radiotherapy regimens on secreted factors and DC maturation markers.

Having observed alterations in the inflammatory mediators, cytokines and chemokines between the secretome of rectal cancer tissue and non-malignant rectal tissue and the differential effects of radiation on the secretome of non-malignant and malignant rectal tissue, we investigated the effect of the secretome on immune cell function, specifically DC maturation markers. DCs are antigen-presenting cells capable of inducing a T cell response, and maturation levels have been associated with patient survival and response to targeted therapies in CRC patients [38,39]. It has previously been reported that the TME in CRC causes inhibition of DC maturation [40,41]. On the contrary, however, we found upregulation of DC maturation by the rectal cancer TME, which is in line with results published by Morrissey et al. [41]. This finding warrants further investigation since it may have important clinical implications in stratifying patients for whom immunotherapy may offer a clinical benefit. We demonstrated a significant increase in CD80 and PD-L1 on the DCs exposed to the irradiated rectal cancer secretome compared to the mock-irradiated rectal cancer secretome. CD83 was significantly higher on DCs exposed to the mock-irradiated rectal cancer secretome compared to the mock-irradiated normal rectal secretome. Our results are in line with a study by Kulzer et al. where the supernatants of irradiated SW480 colon cancer cells resulted in significant enhancement of DC maturation markers compared to supernatants from mock-irradiated SW480 cells [42]. However, our study presents results from a more translational model system as we used human ex vivo explants. We found similar results in the unstimulated setting without LPS whereby TCM from the irradiated rectal cancer secretome had the most potent effect on DC maturation (Figure S1).

Given the observed differences in the protein secretome of the microenvironments investigated and the differential response of DCs to these microenvironments, we conducted a correlation analysis to identify any relationship between secreted factors and DC maturation markers. In the irradiated normal rectal tissue, there was a significant inverse correlation between IL-10 and CD80. IL-10 has been previously shown to have an immunosuppressive role on circulating DCs in patients with hepatocellular carcinoma [43]. While in the rectal cancer TME, CD80 levels on DCs correlated positively with Flt-1 and inversely with IL-27. VEGF is known to adversely affect DC maturation [44], and blockade of Flt-1 on DCs curtails this effect [45]. It may be possible that VEGF is binding to elevated levels of Flt-1 in the TME and is therefore unavailable to exert inhibitory effects on DC maturation. IL-27 has previously been reported to upregulate PD-L1 on DCs in the absence of DC maturation and is accompanied by a decreased capacity to stimulate T cells [46]. There was a positive correlation between CD83 and SAA in the mock-irradiated rectal cancer TME. SAA has been shown to enhance DC maturation [47]. In the irradiated rectal cancer, TME CD80 correlated with ICAM-1 and CD11c correlated with IL-1RA. These results indicate that the response of DCs to their microenvironment differs between normal and malignant rectal tissue and that radiation further modifies this response. It is possible that the interplay between the relative concentration of cytokines in the distinct microenvironments examined exerts differential effects on DC maturation.

Overweight and obese rectal cancer patients have been reported to have poorer outcomes than their counterparts of a healthy weight [48,49]. To identify if obesity had a direct effect on secreted factors within the TME and subsequent immune response, we correlated body composition parameters with secreted factors and markers of DC maturation. We found positive correlations between VFA and CCL20 in the mock-irradiated TME and VFA and CCL20 and IL-1RA in the irradiated TME. Moreover, intermuscular fat, a marker which is associated with insulin resistance and metabolic dysfunction [50], was correlated with CCL20, IL-1RA and VEGF-A. There were positive correlations between levels of the phenotypic marker CD11c on the DCs treated with TCM from irradiated rectal cancer tissue and VFA and intermuscular fat. These data suggest that obesity status may directly alter the milieu of inflammatory proteins in the TME, and these factors may be differentially altered by radiation in obese individuals. Further exploration of the relationship between obesity and the inflammatory microenvironment of rectal cancer patients may reveal novel therapeutic targets.

Strengths of this study include the use of whole biopsies as experimental models since these models recapitulate the microenvironment and the 3-D architecture of human tumours and normal tissue. However, a limitation associated with these models is their long-term viability ex vivo (up to 72 h). Our study was limited to the use of a single fraction of a clinically relevant dose of radiation, though it must be acknowledged that repeated fractions of radiation may induce different responses. Future study utilizing biopsy specimens obtained at surgical resection from both treatment-naïve patients and from patients that received a full course of neo-CRT would provide important information on the effect of neo-CRT on the inflammatory milieu and its interaction with the innate immune system.

We have conducted, for the first time, a comprehensive profile of the rectal cancer and normal rectal secretome and demonstrated differential expression of cytokines, chemokines and inflammatory markers in the secretome of rectal cancer tissue compared to normal rectal tissue. This study is novel as it assesses the secreted proteins in the microenvironment of malignant and non-malignant rectal tissue, therefore identifying aberrant expression of factors in the immediate vicinity of the tumour, while most studies to date investigate the circulating levels of such inflammatory proteins. Investigating the secretome of the tissue offers insight into the locally acting factors involved in the disease process as well as potentially identifying factors involved in the disease at an early stage. Furthermore, we have shown that a single fraction of a clinically relevant dose of radiation exerts differential effects on the secretome of normal rectal tissue compared to malignant rectal tissue. Finally, DC maturation status is enhanced by the irradiated rectal cancer secretome compared to the irradiated normal rectal secretome, indicating an immunogenic stimulatory effect of radiation in the rectal cancer secretome that could potentially be harnessed to improve therapeutic response.

## 4. Materials and Methods

### 4.1. Ethics Statement

Ethical approval was granted by the St. James’s Hospital/AMNCH Research Ethics Committee (ref: 2011/43/02). All procedures followed were in accordance with the Declaration of Helsinki and GDPR. Informed written patient consent was obtained for the use of patient tissue and data in this study. Patient data were pseudo-anonymised prior to sample access.

### 4.2. Patient Recruitment

Patients undergoing diagnostic endoscopy for rectal cancer and lower gastrointestinal investigations were prospectively recruited to this study between January 2018 and November 2018. Biopsies were obtained from treatment-naïve patients at diagnostic endoscopy. A total of 12 patients with histologically confirmed rectal cancer and 8 patients that did not have cancer (normal, non-cancer controls) were recruited to the study. All clinical and pathological data were obtained following written informed consent. Clinical data were obtained from patient records. Histological confirmation of tumour tissue and non-malignant tissue in patient diagnostic biopsies was performed by a pathologist using routine haematoxylin and eosin staining. Tumour regression score (TRS) was assigned by a pathologist following surgery in all patients receiving neoadjuvant treatment. VFA, subcutaneous fat area, intermuscular fat and skeletal muscle were calculated from a pre-operative diagnostic computed tomography scan by an experienced radiologist. Patients with a VFA greater than 163.8 cm^2^ (males) and 80.1 cm^2^ (females) were classified as obese [51]. Patient characteristics are outlined in Supplementary Table S1.

### 4.3. Generation of Tumour Conditioned Media and Normal Conditioned Media

TCM from rectal cancer biopsies and NCM from non-cancer control biopsies were generated by rinsing the biopsy four times in PBS supplemented with 1% penicillin–streptomycin, 1% Fungizone and 0.1% gentamicin. The biopsy was then placed in 1 mL of M199 media supplemented with 10% FBS, 1% penicillin–streptomycin, 1% Fungizone, 0.1% gentamicin and 1 μg/mL insulin. Biopsies were incubated for 80 min at 37 °C and 5% CO_2_. Following 80 min incubation, biopsies were either mock-irradiated (0 Gy) or irradiated with 1.8 Gy radiation using an Xstrahl RS225 x-irradiator at a dose rate of 1.73 Gy/min (XSTRAHL, Surrey, UK). Biopsies were then incubated for 24 h at 37 °C and 5% CO_2_. Following 24 h incubation the media were harvested and stored in a 2 mL cryotube at −80 °C until required. The biopsies were snap-frozen in liquid nitrogen and stored at −80 °C. NCM/TCM was diluted 1:1 with M199, and dilutions below refer to dilutions of this.

### 4.4. Mesoscale Discovery 54-Plex ELISA

To assess angiogenic, vascular injury, inflammatory, cytokine and chemokine secretions, a 54-plex ELISA kit spread across 7 plates was used (Meso Scale Diagnostics, Rockville, MD, USA). The 54-multiplex kit was used to quantify the secretions of CRP, CCL11 (Eotaxin), CCL26 (Eotaxin-3), FGF(basic), Flt-1, GM-CSF, ICAM-1, IFN-γ, IL-10, IL-12/IL-23p40, IL-12p70, IL-13, IL-15, IL-16, IL-17A, IL-17A/F, IL-17B, IL-17C, IL-17D, IL-1RA, IL-1α, IL-1β, IL-2, IL-21, IL-22, IL-23, IL-27, IL-3, IL-31, IL-4, IL-5, IL-6, IL-7, IL-8, IL-8 high abundance (HA), IL-9, CXCL10 (IP-10), CCL2 (MCP-1), CCL13 (MCP-4), CCL22 (MDC), CCL3 (MIP-1α), CCL4 (MIP-1β), CCL20 (MIP-3α), PlGF, SAA, CCL17 (TARC), Tie-2, TNF-α, TNF-β, TSLP, VCAM-1, VEGF-A, VEGF-C and VEGF-D from NCM and TCM from each individual biopsy per patient. All assays were run as per manufacturer’s recommendations, with an alternative protocol overnight supernatant incubation being used for all assays except vascular injury and angiogenesis assays. Secretion data for all factors were normalised appropriately to rectal tissue protein content using the BCA assay (Pierce).

### 4.5. Dendritic Cell Isolation and Culture

Human monocyte-derived immature DCs were generated from peripheral blood mononuclear cells obtained from buffy coat preparations (National Blood Centre, St. James’s Hospital, Dublin, Ireland) by density gradient centrifugation (Lymphoprep) as described [38,52]. Briefly, monocytes were isolated by positive selection using anti-CD14 magnetic microbeads as described by the manufacturer (Miltenyi Biotec, Bergisch Gladbach, Germany) and seeded at a density of 1 × 10^6^ cells/mL in 6-well plates in 3 mL of RPMI-1640 medium containing 10% defined low-endotoxin HyClone FBS (Thermo Fischer Scientific, Waltham, MA, USA), 1% penicillin-streptomycin, 1% Fungizone, human granulocyte macrophage colony-stimulating factor (50 ng/mL) (Immunotools, Friesoythe, Germany) and human IL-4 (70 ng/mL) (Immunotools, Friesoythe, Germany) in a humidified atmosphere with 5% CO_2_ at 37 °C. Cells were fed at day 3 by replacing half the medium made up with fresh cytokines. At day 6, CD11c^+^ cells exhibited an immature DC phenotype capable of upregulating maturation markers following LPS activation.

### 4.6. Stimulation of Monocyte-Derived Dendritic Cells

Freshly generated DCs were plated in 96-well plates at 2 × 10^5^ cells in 200 μL RPMI 1640 media supplemented with 10% defined low-endotoxin HyClone FBS (Thermo Fisher Scientific, Waltham, MA, USA) and stimulated with a 1:2 dilution of TCM or NCM, or matched background media controls, for 4–5 h before exposure to 10 μg/mL of ultrapure TLR4 agonist *Escherichia coli* lipopolysaccharide (LPS-EB; Invivogen) overnight. Supernatants were harvested and frozen, and cells were assessed for expression of surface markers as described below.

### 4.7. Flow Cytometry

DCs were stained with the following antibody panel: phycoerythrin (PE)- anti-CD80 (2D10), PerCP-Cy5.5- anti-CD86 (IT2.2), Pe-Cy7- anti-CD83 (HB15), Brilliant Violet 421- anti-PD-L1 (29E.2A3), Brilliant Violet 510- anti-CD11c (3.9), allophycocyanin (APC)- anti-CD54 (HA58) and APC-Cy7- anti-HLA-DR (L243) (Biolegend, San Diego, CA, USA). Samples were acquired on DAKO CyAn ADP flow cytometer (Beckman Coulter, Brea, CA, USA) with compensation performed with positive and negative compensation beads (BD Biosciences, San Jose, CA, USA). Gating on and analysis of CD11c^+^ cells was performed using FlowJo software (Tree Star Inc., Ashland, OR, USA).

### 4.8. Statistical Analysis

GraphPad Prism 9 software was used to perform statistical analysis. All data were expressed as mean ± SEM. Statistical tests used are indicated in each figure legend. Correlation was measured using Spearman correlation coefficient. Statistical significance was considered at *p* < 0.05.

## 5. Conclusions

This study quantified the secretion of 54 proteins from normal rectal tissue and rectal cancer tissue and examined the effect of a clinically relevant dose of radiation on the levels of protein secretions. We have shown that the cancer secretome is more inflammatory than the normal rectal secretome with 19 factors secreted at significantly higher levels from the rectal cancer TME. A single fraction of a clinically relevant dose of radiation alters the inflammatory milieu of the normal but not the rectal cancer microenvironment. We also demonstrated that the irradiated rectal cancer microenvironment induced the most potent effect on stimulating DC maturation, suggesting that tumour irradiation does not have a negative impact on the ability of the rectal cancer TME to induce an anti-tumour immune response. Correlations were found between secretions of inflammatory mediators and clinical characteristics, including obesity status, suggesting that obesity may directly alter protein secretions from the TME.

## Figures and Tables

**Table 1:** Correlation between DC maturation markers and secreted factors in the microenvironment of irradiated normal rectal tissue, i.e., Normal 1.8 Gy.

DC Marker	Secreted Factor	r Value	*p* Value	*n*
**CD80**	IL-10	−0.8571	0.01	8

**Table 2:** Correlation between DC maturation and phenotypic markers and secreted factors in the microenvironment of rectal cancer tissue.

CANCER 0 GY					CANCER 1.8 GY				
DC Marker	Secreted Factor	r Value	*p* Value	*n*	DC Marker	Secreted Factor	r Value	*p* Value	*n*
CD80	Flt-1	0.7857	0.04	7	CD80	ICAM-1	0.8214	0.03	7
CD80	IL-27	−0.8214	0.03	7	CD11c	IL-1RA	0.7619	0.03	8
CD83	SAA	0.7857	0.04	7					

**Table 3:** Correlation between DC maturation and phenotypic markers and secreted factors from DCs treated with NCM.

NORMAL 1.8 GY				
DC Marker	Secreted Factor	r Value	*p* Value	*n*
CD83	ICAM-1	−0.7857	0.02	8
CD11c	ICAM-1	−0.7857	0.02	8

**Table 4:** Correlation between DC maturation and phenotypic markers and secreted factors from DCs treated with TCM.

CANCER 0 GY					CANCER 1.8 GY				
DC Marker	Secreted Factor	r Value	*p* Value	*n*	DC Marker	Secreted Factor	r Value	*p* Value	*n*
PD-L1	TNF-α	0.8333	0.01	8	CD86	VEGF-C	−0.9276	0.01	6
					CD11c	VEGF-C	−0.8857	0.03	6

**Table 5:** Correlation between body composition parameters and factors secreted from mock-irradiated rectal cancer tissue, i.e., Cancer 0 Gy.

Body Composition Parameter	Secreted Factor	r Value	*p* Value	*n*
Skeletal muscle	Flt-1	−0.6273	0.04	11
Skeletal muscle	IL-12/IL-23p40	0.6573	0.02	12
Skeletal muscle	IL-1α	0.5874	0.04	12
Skeletal muscle	VEGF-A	0.6224	0.03	12
Visceral fat area	CCL20	0.6783	0.01	12

**Table 6:** Correlation between body composition parameters and factors secreted from irradiated rectal cancer tissue, i.e., Cancer 1.8 Gy.

Body Composition Parameter	Secreted Factor	r Value	*p* Value	*n*
Skeletal muscle	Flt-1	−0.7182	0.01	11
Skeletal muscle	VEGF-D	−1	0.01	5
Intermuscular fat	CCL20	0.7133	0.01	12
Intermuscular fat	VEGF-A	0.6084	0.03	12
Intermuscular fat	IL-1RA	0.6084	0.03	12
Visceral fat area	CCL20	0.6643	0.02	12
Visceral fat area	IL-1RA	0.6503	0.02	12

## Data Availability

The data presented in this study are available upon reasonable request from the corresponding author. The data are not publicly available as there are restrictions on data processing in line with participant consent.

## Supplementary Materials

The following are available online at https://www.mdpi.com/2072-6694/13/3/571/s1, Figure S1: The effect of NCM and TCM on unstimulated dendritic cells, Figure S2: Correlations between secreted factors from mock-irradiated rectal cancer tissue (Cancer 0 Gy) and body composition parameters, Figure S3: Correlations between secreted factors from irradiated rectal cancer tissue (Cancer 1.8 Gy) and body composition parameters, Table S1: Patient characteristics.

## Abbreviations

CRC	colorectal cancer
DC	dendritic cell
LPS	lipopolysaccharide
NCM	normal conditioned media
neo-CRT	neoadjuvant chemoradiation
TCM	tumour conditioned media
TME	tumour microenvironment
T reg	regulatory T cell
TLR	toll-like receptor
TRS	tumour regression score
VFA	visceral fat area
