# Supplementary material for: The Protein Secretome Is Altered in Rectal Cancer Tissue Compared to Normal Rectal Tissue, and Alterations in the Secretome Induce Enhanced Innate Immune Responses
Source: Cancers (Basel). 2021 Feb 2;13(3):571. doi: 10.3390/cancers13030571 (PMC7867296; doi:10.3390/cancers13030571)
Supplement: Supplementary file 1 [file cancers-13-00571-s001.pdf]

cancers

MDPI

Supplementary materials

The Protein Secretome Is Altered in Rectal Cancer Tissue Compared to Normal Rectal Tissue, and Alterations in the Secretome Induce Enhanced Innate Immune Responses

Aisling B. Heeran [1], Margaret R. Dunne [1], Maria E. Morrissey [1], Croí E. Buckley [1], Niamh Clarke [1], Aoife Cannon [1], Noel E. Donlon [1], Timothy S. Nugent [1], Michael Durand [2], Cara Dunne [2], John O. Larkin [2], Brian Mehigan [2], Paul McCormick [2], Niamh Lynam-Lennon [1] and Jacintha O'Sullivan [1,*]

[1] Trinity St. James's Cancer Institute, Trinity Translational Medicine Institute, Department of Surgery, Trinity College Dublin and St. James's Hospital, Dublin 8, D08 W9RT, Ireland; heerana@tcd.ie (A.B.H.); dunnem12@tcd.ie (M.R.D.); miamorrissey@gmail.com (M.E.M.); bucklecr@tcd.ie (C.E.B.); clarken1@tcd.ie (N.C.); cannona@tcd.ie (A.C.); donlonn@tcd.ie (N.E.D.); nugentti@tcd.ie (T.S.N.); lynamlen@tcd.ie (N.L.-L.)

[2] GEMS, St. James's Hospital, Dublin 8, D08 NHY1, Ireland; durandm@tcd.ie (M.D.); cardunne@stjames.ie (C.D.); jlarkin@stjames.ie (J.O.L.); bmehigan@stjames.ie (B.M.); pmccormick@stjames.ie (P.M.)

* Correspondence: osullij4@tcd.ie; Fax: +353-(0)18964122

Supplementary materials

Citation: Heeran, A.B.; Dunne, M.R.; Morrissey, M.E.; Buckley, C.E.; Clarke, N.; Cannon, A.; Donlon, N.E.; Nugent, T.S.; Durand, M.; Dunne, C.; et al. The Protein Secretome is Altered in Rectal Cancer Tissue Compared to Normal Rectal Tissue and Alterations in the Secretome Induce Enhanced Innate Immune Responses. *Cancers* **2021**, *13*, 571. https://doi.org/10.3390/cancers13030571

Received: 12 January 2021
Accepted: 25 January 2021
Published: 2 February 2021

Publisher's Note: MDPI stays neutral with regard to jurisdictional claims in published maps and institutional affiliations.

Copyright: © 2021 by the authors. Licensee MDPI, Basel, Switzerland. This article is an open access article distributed under the terms and conditions of the Creative Commons Attribution (CC BY) license (http://creativecommons.org/licenses/by/4.0/).

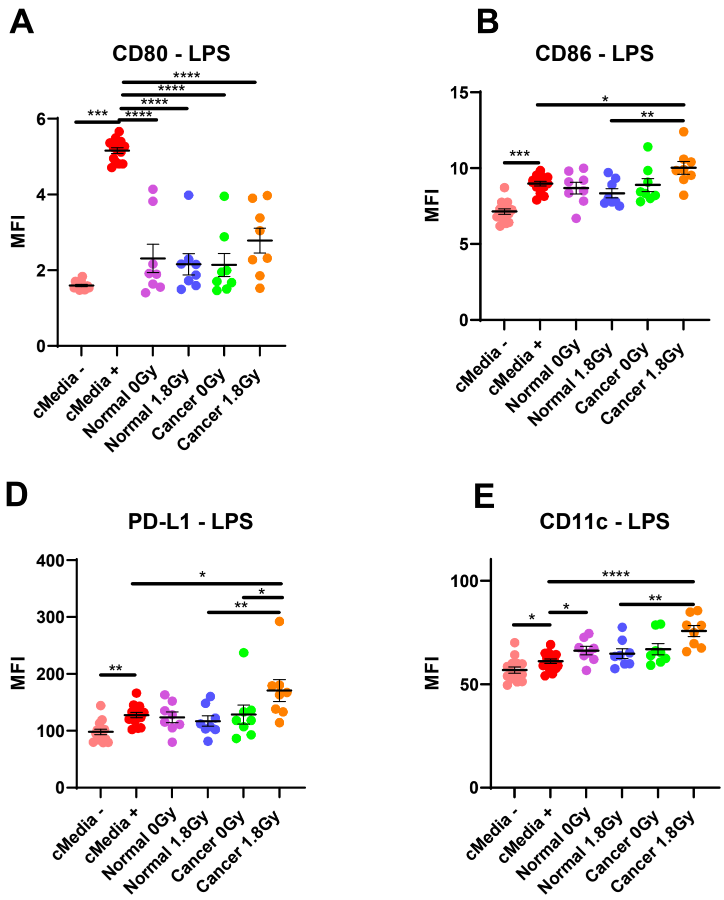

cMedia-: M199 without LPS stimulation
cMedia+: M199 with LPS stimulation

Figure S1. The effect of NCM and TCM on unstimulated dendritic cells. (**A**) Expression levels of CD80 were significantly inhibited by NCM and TCM from both mock-irradiated and irradiated biopsies compared to LPS-induced expression of CD80. (**B**) Expression of CD86 was significantly elevated on DCs treated with TCM from irradiated rectal cancer biopsies compared to LPS-induced expression of CD86. TCM from irradiated rectal cancer biopsies induced significantly higher expression of CD86 compared to NCM from irradiated normal rectal biopsies. (**C**) TCM from irradiated rectal cancer tissue induced higher expression of CD83 compared to LPS-induced expression of CD83. (**D**) PD-L1 expression was significantly elevated on DCs treated with TCM from irradiated rectal cancer biopsies compared to LPS-induced expression of PD-L1. TCM from irradiated rectal cancer tissue enhanced expression of PD-L1 compared to TCM from mock-irradiated rectal cancer tissue or NCM from irradiated normal rectal tissue. (**E**) CD11c levels were significantly elevated on DCs treated with TCM from irradiated rectal cancer tissue and NCM from mock-irradiated rectal cancer tissue compared to LPS-induced expression of CD11c. TCM from irradiated rectal cancer tissue induced greater expression of CD11c compared to NCM from irradiated normal rectal tissue. All data expressed as mean ± SEM. Statistical analysis was performed using a Wilcoxon signed-rank test when comparing the same tissue type i.e. Cancer 0 Gy vs Cancer 1.8 Gy and Mann Whitney U-test when comparing different tissue types and comparing to media control. n=14 for cMedia, n=8 for normal and cancer, ****p<0.0001, ***p<0.001, **p<0.01, *p<0.05.

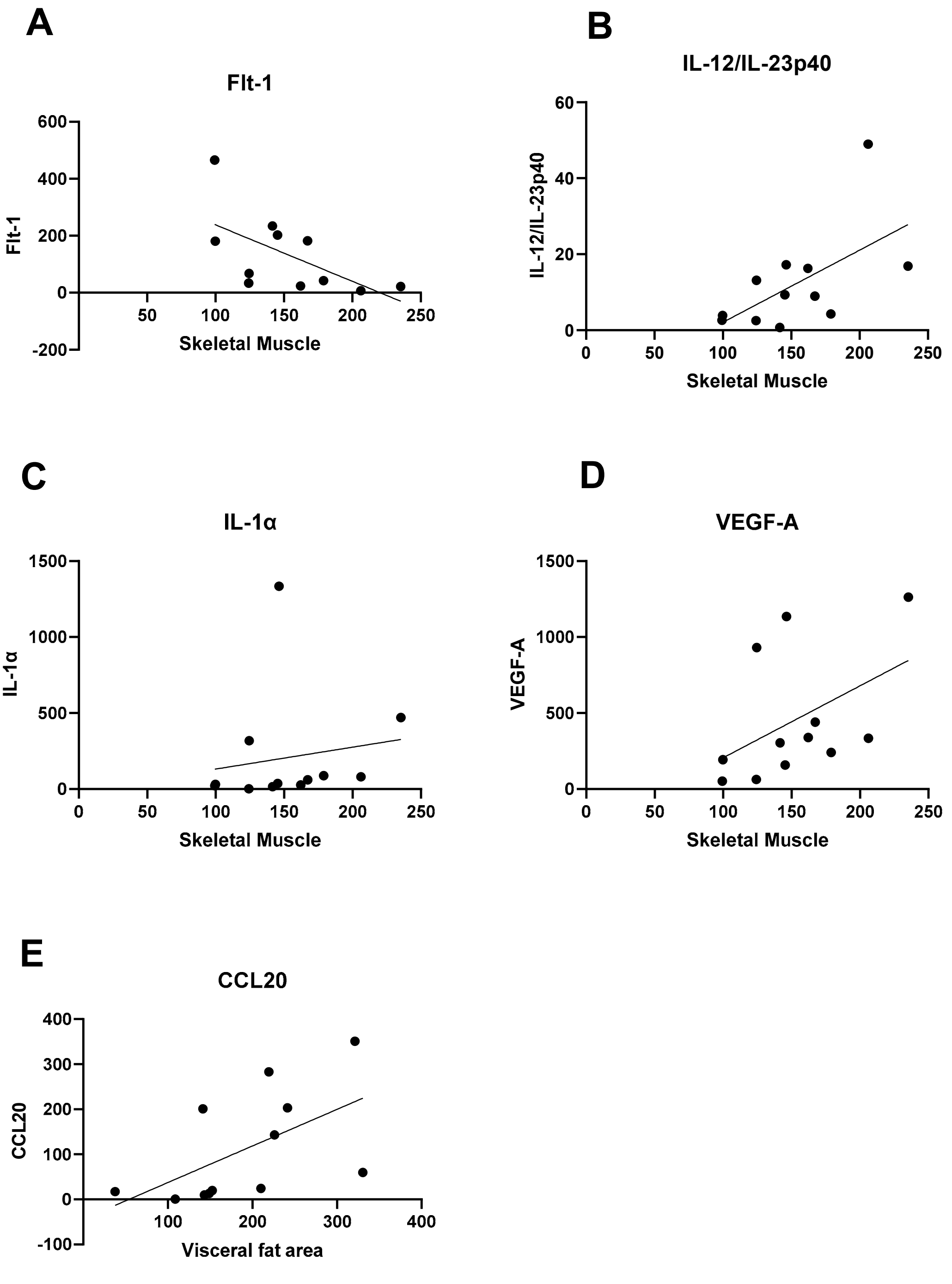

Figure S2. Correlations between secreted factors from mock-irradiated rectal cancer tissue (Cancer 0 Gy) and body composition parameter. (A) There was a significant inverse correlation between skeletal muscle and Flt-1 (R=-0.6273, p=0.04). (B) There was a significant correlation between skeletal muscle and IL-12/IL-23p40 (R=0.6573, p=0.02). (C) IL-1α was significantly correlated with skeletal muscle (R=0.5874, p=0.04). (D) VEGF-A was significantly correlated with skeletal muscle (R=0.6224, p=0.03). (E) Visceral fat area was correlated with CCL20 (R=0.6783, p=0.01). Correlation analysis was performed using Spearman correlation coefficient. n=12, n=11 for Flt-1.

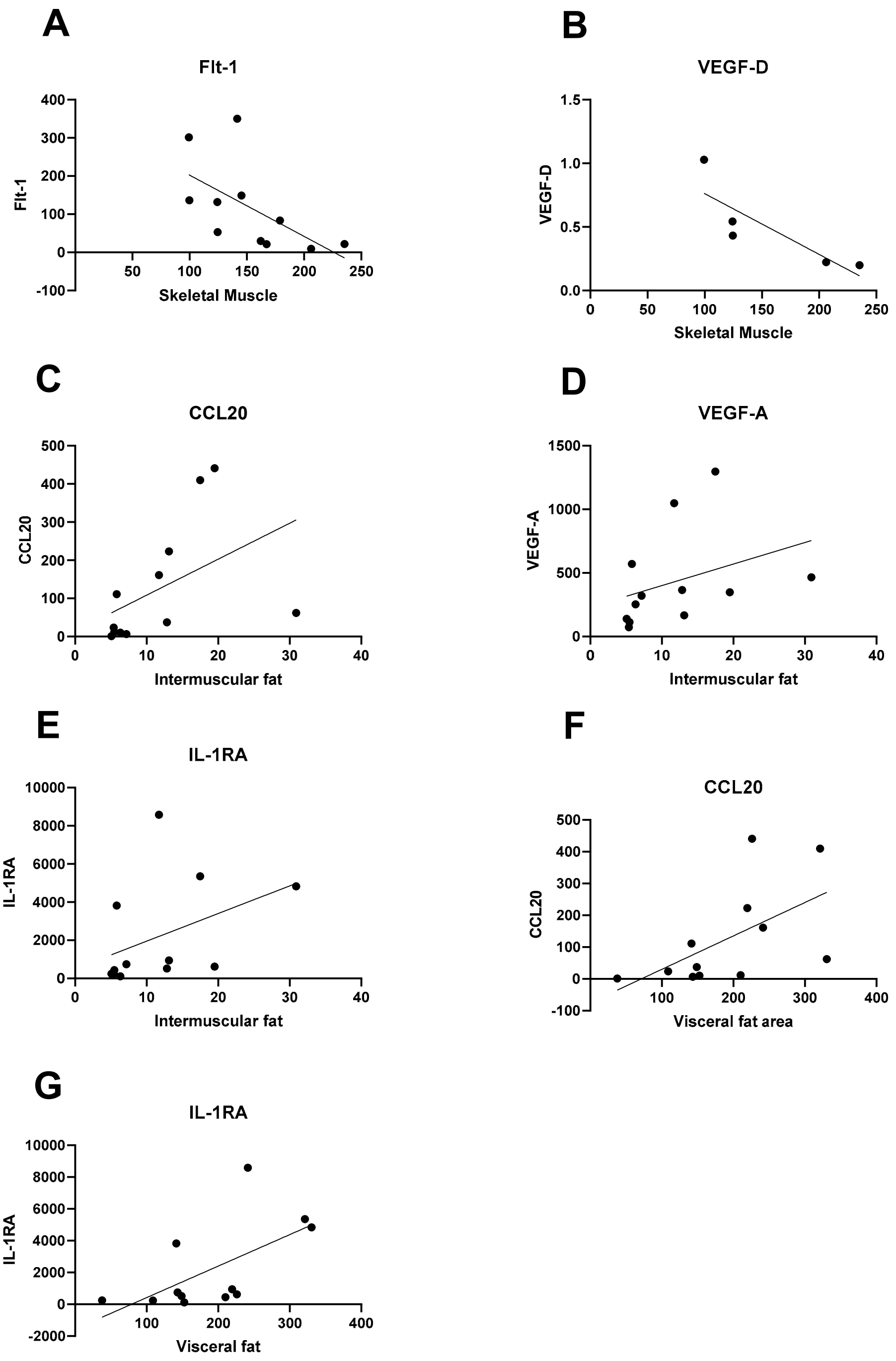

Figure S3. Correlations between secreted factors from irradiated rectal cancer tissue (Cancer 1.8 Gy) and body composition parameters (**A**) There was a significant inverse correlation between skeletal muscle and Flt-1 (R=-0.7182, *p*=0.01). (**B**) There was a significant inverse correlation between skeletal muscle and VEGF-D (R=-1, *p*=0.01). (**C**) CCL20 was significantly correlated with intermuscular fat (R=0.7133, *p*=0.01). (**D**) VEGF-A was significantly correlated with intermuscular fat (R=0.6084, *p*=0.03). (**E**) Intermuscular fat was correlated with IL-1RA (R=0.6084, *p*=0.03). (F) CCL20 correlated with visceral fat area (R=0.6643, *p*=0.02) and (**G**) There was a significant correlation between IL-1RA and visceral fat area (R=0.6503, *p*=0.02). Correlation analysis was performed using Spearman correlation coefficient. n=12, n=11 for Flt-1.

Table S1. Patient characteristics.

			**Percent (%)**
**Age (rectal cancer patients)**	Mean ± SD	65.16 ± 10.14	
	Range	53-89	
**Age (control patients)**	Mean ± SD	58.75 ± 11.42	
	Range	44-76	
**Gender (rectal cancer patients)**	Male (n)	8	66.67
	Female (n)	4	33.33
**Gender (control patients)**	Male (n)	3	37.5
	Female (n)	5	62.5
**Obesity status (visceral fat area)**	Obese	8	66.67
	Non obese	4	33.33
**Histology**	Adenocarcinoma (n)	12	100
**Stage of differentiation**	Moderate (n)	12	100
**T stage**	T1 (n)	1	8.33
	T2 (n)	2	16.67
	T3 (n)	8	66.67
	T4 (n)	1	8.33
**N stage**	N0 (n)	6	50
	N1 (n)	5	41.67
	N2 (n)	1	8.33
**M stage**	M0 (n)	11	91.67
	M1 (n)	1	8.33
**Neoadjuvant CRT**	Received neo-CRT (n)	6	50
**Neoadjuvant CT**	Received neo-CT only (n)	1	8.33
**Neoadjuvant RT**	Received neo-RT only (n)	1	8.33
**Surgery** [a]	Received surgery (n)	11	91.67
**TRS** [b]	0 (n)	2	28.57
	1 (n)	3	42.85
	2 (n)	1	14.28
	3 (n)	1	14.28

Abbreviations; CRT; chemoradiotherapy, CT; chemotherapy, RT; radiotherapy, TRS; tumour regression score [a] One patient was unsuitable for surgery [b] TRS available for 7 patients, percent expressed as total with TRS score.
